# A Measurement System for the Environmental Load Assessment of a Scale Ship Model—Part II

**DOI:** 10.3390/s23073415

**Published:** 2023-03-24

**Authors:** Anna Miller, Andrzej Rak

**Affiliations:** Department of Ship Automation, Electrical Engineering Faculty, Gdynia Maritime University, 81-87 Morska Str., 81-225 Gdynia, Poland

**Keywords:** wave height measurement, wave spectra estimation, microprocessor devices, environmental disturbances, modeling of the ship motion process, ship scale model

## Abstract

In the process of ship motion control system design, it is necessary to take into account the impact of environmental disturbances such as winds, waves and sea currents. The commonly used representatives of wave influences in this area are the unidirectional wave power spectral density functions describing sea waves of different form: long-crested, fully developed waves, developing wind waves or multi-modal waves (e.g., with swell). The existing standard PSD models describe the surge of open sea or ocean. However, they are inadequate in the case of control system testing of scale ship models for sailing in open water areas such as lakes or test pools. This paper presents a study of wind-generated wave PSD estimations for a small lake used as a test area for free-running scale ships. The publication provides a brief overview of the wave spectral density functions commonly used for control system design. A measurement instrument using the idea of a water-induced variable capacitance that works synchronously with the wind sensors is also described. The process of collected data analysis is presented. As a result of the study, a series of empirical spectral density functions of lake waves for different wind speeds are obtained. They correspond to the rescaled, two-parametric ITTC model.

## 1. Introduction

This work deals with modeling of the effects of environmental disturbances on ship models, built in 1:24 scale, sailing on the small Silm Lake near Iława in northern Poland [1]. Both the site and the measurement system used in the study were presented in a twin paper devoted to the wind measurement and analysis [2]. This second part of the publication addresses modeling of the wave load.

During the last decade, development in the field of marine transportation systems has been largely associated with the concept of an autonomous seagoing vessel that would be able to make high seas voyages from port to port without the presence of a crew on board. The intensive research that is being carried out in this area, among other legal, economical and technical factors, also concerns the motion control system of such a vessel. During this research, at the stage of verifying the correct functioning of the control system, tests are often carried out using scale models of the sea-going ships sailing in towing tanks, model basins or in open waters [3,4,5,6,7]. In the latter case, these are usually ponds or small lakes. Thus, there is a need to determine the wave and wind parameters that occur in such water areas, since existing models of environmental disturbances have been constructed on the basis of measurements made over seas and oceans. Most often, these were done in the North Atlantic, which naturally does not match the environmental conditions in inland water areas.

On the other hand, published works concerning lake wave analysis refer to mathematical models such as SWAN or WW3, for example [8,9,10,11]. These models are of a different nature than used to describe the waves loads of a marine vessel. The measurement data for which the cited studies are based on were collected on large bodies of water, such as the American Great Lakes. Therefore, the phenomena occurring there cannot be compared to the processes arising on a lake representing relatively very small waters, which is the subject of this work. During the literature review, no works were found that presented a spectral model of the waves on a small lake of a similar nature to the one under study.

Proposed models of wind and waves acting on scale ships are essential at the stage of simulating control system operation as well as in verification of particular control structures that take inputs from environmental phenomena into account.

As with wind interference, the effect exerted by waves on a ship sailing on the surface of the water can be divided into two components [12]:

- a slowly varying component, loaded with a bias component, which is responsible for the wave drift of the vessel;
- a fast varying component, which consists of irregular oscillations with zero mean value, called wave frequency (WF) motions.

The forces acting on the ship’s hull, the source of which are both of these components, are determined using RAOs (response amplitude operators) [13], or using the linear approximation of the wave force models determined separately for each degree of freedom of the ship [14]. In either case, the input of the particular formula is the amplitude of the wave [15].

The standard source of information about the parameters of an irregular wave is its description in the form of a spectrum. Therefore, the Section 2 of this article presents the commonly used standard forms for spectral description of sea wave height caused by wind. The Section 3 of the paper discusses the technical aspects of measuring wave height on the lake. The Section 4 describes the measurement results obtained, which are then discussed in the Section 5. The article closes with the conclusions obtained as a result of the research.

## 2. Spectral Wave Representation in Estimation of Ship Forces

When testing the robustness and performance of ship motion control systems, one may assume that the vessel is affected by a long-crested irregular sea. Wave elevation can then be defined as a composite of a series of harmonic components in the form of [16]:

(1) $$\xi =\sum_{k=1}^{N}A_{k}\cos\left(\omega_{k}+\epsilon_{k}\right),$$

where $A_{k}$, $\omega_{k}$ and $\epsilon_{k}$ indicate respectively the wave amplitude, circular frequency and random initial phase of its *k* successive harmonic components. The wave amplitude $A_{k}$ can be drawn from the wave spectral density function $S\left(\omega_{k}\right)$ by the relationship:

(2) $$\frac{1}{2}A_{k}^{2}=S\left(\omega_{k}\right)\;\Delta \omega ,$$

where Δω is constant difference between consecutive frequencies. Therefore, Equation (1) can be rewritten as:

(3) $$\xi =\sum_{k=1}^{N}\sqrt{2S\left(\omega_{k}\right)\;\Delta \omega }\;\cos\left(\omega_{k}+\epsilon_{k}\right).$$

This last dependency is commonly used, usually with the additional caveats, for modeling forces of unidirectional waves acting on a ship, as can bee seen for example in [12] or [17]. Therefore, the knowledge of a spectral description of the water area of interest is a key factor.

The sea wave spectra that are most commonly used in context of the design of ship motion control systems have roots in the research of G. Neumann [18], who proposed an empirical single-parameter spectrum in the form:

(4) $$S\left(\omega \right)=C\omega^{-6}\exp\left(-2g^{2}\omega^{-2}V^{-2}\right),$$

where *C* is an empirically determined constant, *g* is acceleration of gravity and *V* denotes the average wind speed. The relationship developed by Neumann was extended by Bretshneider [19], who proposed the formula:

(5) $$S\left(\omega \right)=\frac{5}{16}\omega_{0}^{4}{H_{1/3}}^{2}\omega^{-5}\exp\left(-1.25{\left(\frac{\omega_{0}}{\omega }\right)}^{4}\right),$$

where $\omega_{0}$ is the modal frequency of the spectrum, and $H_{1/3}$ is the significant wave height, defined as the mean value of the one-third highest waves. This spectrum was prepared for the North Atlantic with unlimited fetch and unidirectional (measured at a point) waves without swell.

The significant wave height is the quantity used to describe sea state codes in different weather conditions. This standard scale is presented in Table 1.

Based on the Equation (5), a family of spectra was established. Thus, Pierson and Moskowitz [21] generalized the Bretshneider notation as:

(6) $$S\left(\omega \right)=A\omega^{-5}\exp\left(-B\omega^{-4}\right),$$

and recommended calculating *A* nad *B* according to:

(7) $$A=8.1\times 10^{-3}g^{2}$$

and

(8) $$B=0.74{\left(\frac{g}{V_{19.4}}\right)}^{4}=\frac{3.11}{H_{1/3}^{2}},$$

where $V_{19.4}$ is the wind velocity measured 19.4 m above the sea level. This recommendation is known as one-parameter Pierson–Moskowitz (PM) spectrum. The Bretshneider formula (5) itself is a two-parameter PM spectrum [22]. One of most frequently used member of this spectral family was specified during the ITTC conference, in which the following formulas were recommended for the calculation of *A* and *B* parameters [23]:

(9) $$A=0.0081g^{2}K^{-4}$$

and

(10) $$B=\frac{4A}{H_{1/3}^{2}},$$

where factor *K* may be determined from different measures of wave period. In this context, the most frequently zero-crossing period $T_{z}$ is used through the following expression [24]:

(11) $$K=\frac{T_{z}}{1.771}\sqrt{\frac{g}{H_{1/3}}}.$$

In this research, *A* and *B* factors were estimated based on the mean values of the wave period (${\bar{T}}_{z}$) and significant wave height (${\bar{H}}_{1/3}$) using:

(12) $$A=\frac{173{\bar{H}}_{1/3}^{2}}{{\bar{T}}_{z}^{4}}$$

and

(13) $$B=691{\bar{T}}_{z}^{4}.$$

More mathematical formulas for calculating *A* and *B* of PM family spectra can be found in the 23rd ITTC report [24].

All abovementioned spectra are derived for the fully developed sea, usually based on the wave measurements collected in the North Atlantic. In the late 1960s, the wave spectrum of the undeveloped sea and restricted fetch was formed based on measurements made in the North Sea as part of the JONSWAP (Joint North Sea Wave Project) research project [25]. It is formed from modification of the PM spectrum by adding a factor increasing its “peakedness”:

(14) $$S\left(\omega \right)=S{\left(\omega \right)}_{PM}\gamma^{M}$$

where $S{\left(\omega \right)}_{PM}$ stands for a Pierson–Moskowitz spectrum given by (6), and γ is proposed to be between 1 and 7. However, typically γ=3.3 and *M* is equal to:

(15) $$M=\exp\left(-\frac{1}{2}{\left(\frac{\omega -\omega_{0}}{\sigma \omega_{0}}\right)}^{2}\right)$$

with

(16) $$\sigma =\left\{\begin{array}{cc} 0.07\;\mathrm{for} & \omega \leq \omega_{0}\\ 0.09\;\mathrm{for} & \omega >\omega_{0}. \end{array}\right.$$

As can be seen from the Equations (14)–(16), the modifying function has the shape of an asymmetrical bell curve with a maximum value equal to γ for the modal frequency $\omega_{0}$. Hence, the parameter γ is called the peak enhancement factor (PEF). It is a normally distributed random quantity with a mean value of 3.3 and a variance of 0.62. The first versions of the JONSWAP spectrum included also a dependence of the *A* and *B* parameters on the fetch limits [26].

The group of the state-of-the-art unidirectional wave spectra used in the field of ship motion control systems are supplemented by the two peaks functions describing waves with a low-frequency swell component. The most frequently used in this class are the formulae of Torsethaugen [27], suited to the North Sea conditions, or Ochi–Hubble [28], applicable to storm rise and decay. These two remain outside the scope of this research because swell waves have not been observed on Silm Lake.

## 3. Device for Wave Measurement

As mentioned in the Section 1, the setup of the measurement hardware was described in the twin paper presenting the results of wind modeling [2]. Thus, a reader interested in the wind measurement method used can refer to that paper. Furthermore, details about wave data processing and transmission can be found in Sikora et al. [29].

### Measurement Hardware Characteristics

**Figure 1:**
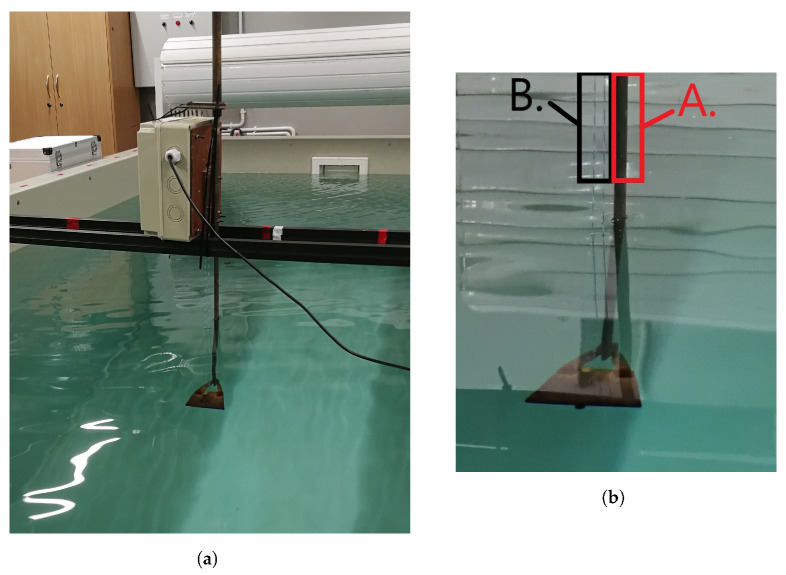
Experiment of wavemeter scaling in a pool: (**a**) wavemeter and (**b**) zoomed-in wavemeter part with two electrodes (A and B) indicated.

The sensor part of the wavemeter is based on the idea of a capacitor with variable capacitance due to floating water level between its electrodes. One electrode is the brass flat bar (Figure 1b—element A), which is also the basic element of the mechanical structure of the sensor. The second electrode (Figure 1b—element B) is composed of a thin copper wire insulated in Teflon coating. The wire is fitted parallel to the bar. The total immersion range of the sensor, 0–500 mm, corresponds to the variance of capacitance ranging from 40 to 400 pF. However, due to the dimensions of hardware brackets installed on the lake and expected wave amplitudes, only the middle 0–300 mm range of the sensor was adopted for use in the experiments.

Verification of the wavemeter measurement accuracy, combined with scaling procedures, was conducted in a test pool (Figure 1). The device was installed in the middle of the tank, and all data were registered in static (calm) water conditions.

**Figure 2:**
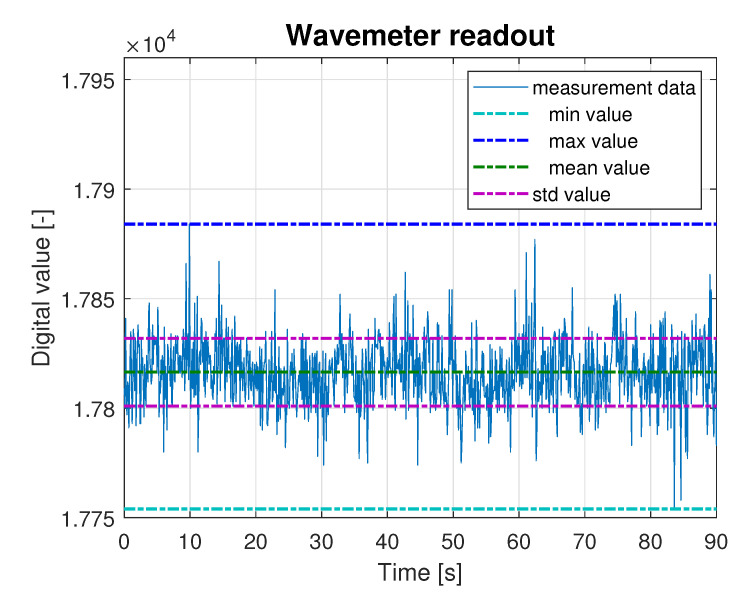
Example of wavemeter readout retrieved from the scaling experiment.

This scaling was performed to verify the linearity of the measurement device characteristics. In each step of the experiment, the wavemeter immersion (Δh) was changed, and the readout was registered with a sampling frequency fs=10 Hz for at least 100 s. Sample recording of digital values involved reading from the wavemeter transducer together with marked minimum, maximum and mean values, with marked signal standard deviation, as presented in Figure 2.

**Figure 3:**
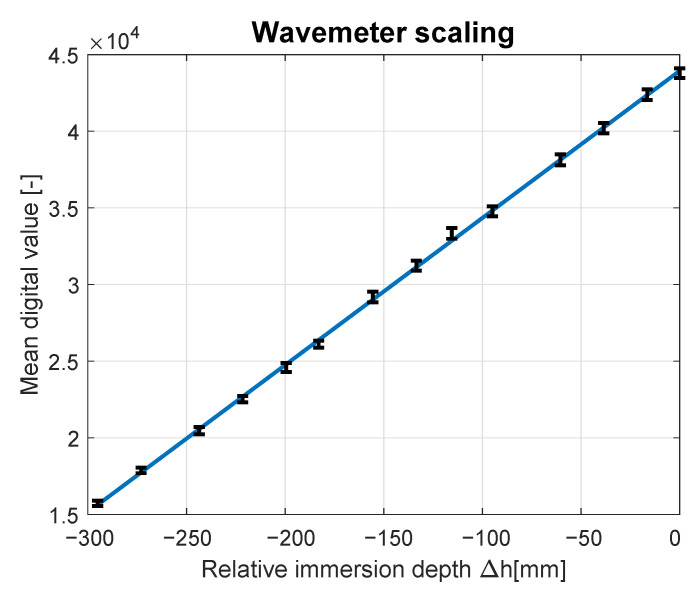
Wavemeter characteristics verification and scaling.

Mean digital values and the corresponding immersion levels for the whole scaling routine are shown in Figure 3. Curve fitting procedure applied to the measured data showed that the static characteristic of the sensor is linear and passes through the measurement error bars. Based on the relation represented by the line marked in blue, conversion Formula (17) was derived. It was used to transform digital values transmitted by the wavemeter DV to the wave elevation *h* in millimeters:

(17) $$h=\frac{DV-43933}{95.92}.$$

Table 2 presents the linear model fit to the recorded digital values DV assessed with the absolute and relative data fit errors. The presented values indicate a high level of compliance between model and data with maximal absolute error ending at 473.40, which is equal to 1.42% of the digital measured value. The mean fit error is equal to 142.38, which corresponds to an average conformity relative error of 0.42%. The obtained results confirm the linear characteristics of the wavemeter for measured waves with an elevation spanning from 0 to 300 mm.

All measurement result statistics for the sensor verification and scaling are summarized in Table 3. Standard deviation of digital signal σ does not exceed 30, which corresponds to 0.12% of the mean digital value. The parameter 3σ indicates the range of water level fluctuations and does not exceed 2.29 mm and has a mean value of 1.71 mm. Therefore, the measurement inaccuracy arising in the presented hardware is negligibly small. The load of wave with the amplitude on the order of millimeters for the ship model built in 1:24 scale is practically imperceptible and small compared with the wave load of several centimeters for a full-scale sea-going vessel.

## 4. Results

The measurement data outlined and analyzed in this section were selected from the set gathered during several measurement sessions held on the Silm Lake, near Iława, Poland, in the period 2019–2022. They were registered using methods stated in Section 2 and measurement equipment described in Section 3. The presented results concern analyzes of five chunks of data collected for wind forces according to the Beaufort wind scale (BFT), ranging from 4 to 9 BFT in the ship scale model. The samples were selected in order to reduce processed data to the cases when the nature of the wind corresponds, for a sufficiently long time, to the conditions distinctive for the particular BFT value. Data for each wind BFT force were examined according to the following scheme:

- raw data extraction and calculation of the deviations from the slow-changing component;
- frequency calculation of mean value;
- computation of the wave significant height and estimation of their probability distribution functions;
- analysis of the correlation of wind speed and wave height as well as wave spectrum assessment.

Consecutive stages of data examination are discussed in the following subsections. Furthermore, to keep the reference to the corresponding open sea phenomena, measured wind speed in all experiments was converted to the force in the BFT, adjusted to the dimensions of the ship model according to the equation:

(18) $$BFT=1.42{\left(\sqrt{su}\;V_{w}\right)}^{1.83/3}$$

where scl = 1:24 is the ship model scale, and $V_{w}$ is the measured wind speed. This conversion is described in more detail in [2]. Similarly, all linear dimensions, particularly wave amplitudes, ought to be scaled up by the factor su=1/scl=24.

### 4.1. Wind Force and Wave Height Correlation

**Figure 4:**
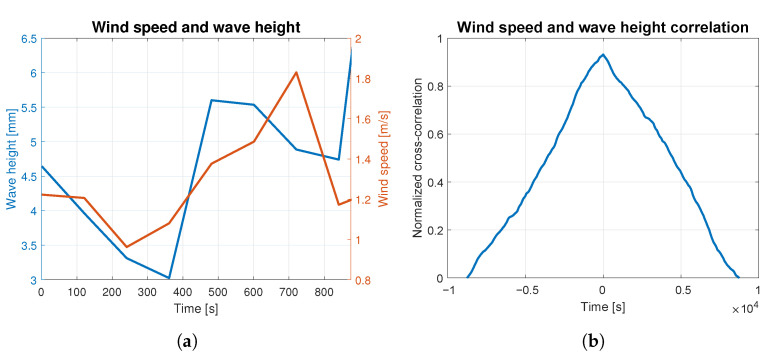
Wind speed and wave height correlation for 4 BFT: (**a**) wind speed and wave height measurements; (**b**) wind speed and wave height correlation.

**Figure 5:**
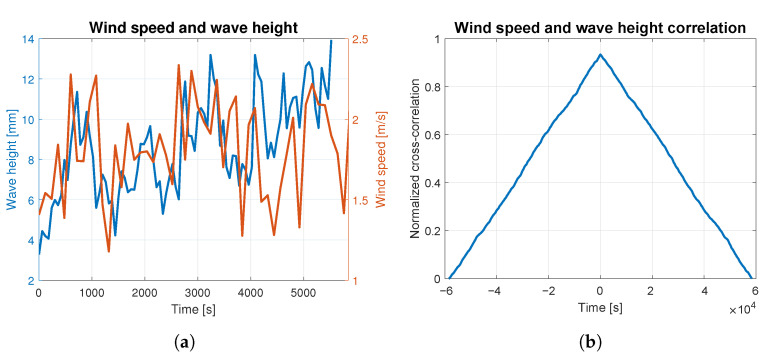
Wind speed and wave height correlation for 5 BFT: (**a**) wind speed and wave height measurements; (**b**) wind speed and wave height correlation.

**Figure 6:**
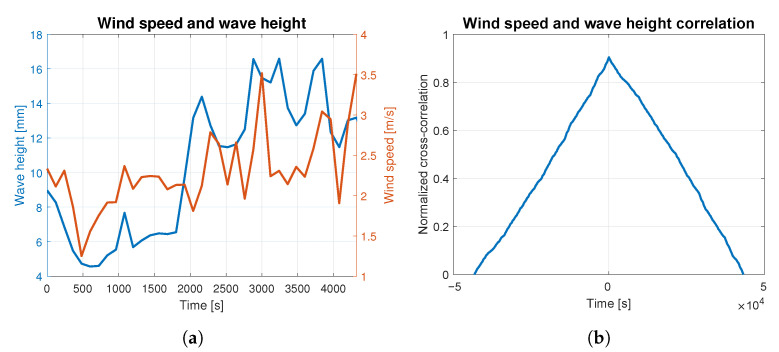
Wind speed and wave height correlation for 6 BFT: (**a**) wind speed and wave height measurements; (**b**) wind speed and wave height correlation.

**Figure 7:**
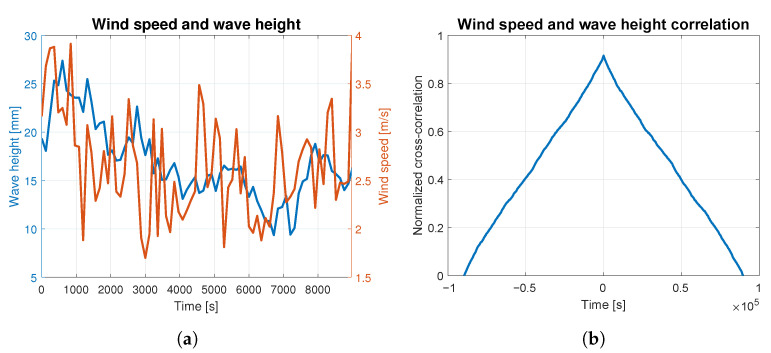
Wind speed and wave height correlation for 7 BFT: (**a**) wind speed and wave height measurements; (**b**) wind speed and wave height correlation.

**Figure 8:**
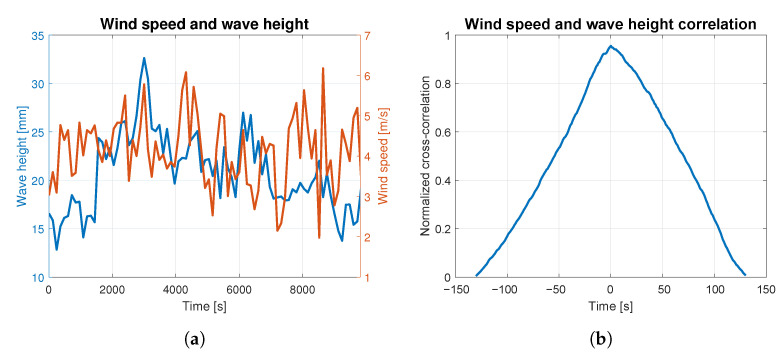
Wind speed and wave height correlation for 9 BFT: (**a**) wind speed and wave height measurements; (**b**) wind speed and wave height correlation.

In order to assess to what extent the waves were caused by the wind, the correlation analysis was carried out for both phenomena. The average wind speed and wave height are presented together in Figure 4a, Figure 5a, Figure 6a, Figure 7a and Figure 8a. The signals are represented by the red and blue lines, respectively. Qualitative convergence of data is observed in all five analyzed cases. Therefore, the waves may be considered as the wind-induced waves.

In order to show the quantitative relationship between wind and waves, normalized correlation between these pairs of recorded time courses was computed according to the formula:

(19) $${\hat{R}}_{xy}\left(m\right)=\{\begin{array}{cc} \frac{1}{N-|m|}\sum_{n=0}^{N-m-1}x_{n+m}y{*}_{n} & \mathrm{for}\;m\geq 0,\\ {\hat{R}}_{yx}^{*}\left(-m\right), & \mathrm{for}\;m<0. \end{array}$$

The normalized correlation function for zero shift takes values equal to: 0.93, 0.92, 0.90, 0.91 and 0.95 for wind-generated waves of 4, 5, 6, 7 and 9 BFT, respectively. Graphs of correlation functions presented in Figure 4b, Figure 5b, Figure 6b, Figure 7b and Figure 8b are qualitatively similar to equilateral triangles. This indicates the high dependency of the wind speed and WF component of the waves, to the order of 90%.

The obtained results show that the examined waves are wind-induced waves. Therefore, significant ’lake’ wave analysis was conducted using the same methods as for ’sea’ wind-induced waves. Scaled to the training ship dimensions, the significant waves in the lake are presented in Section 4.2.

### 4.2. Significant Height Analysis of the Wind Induced Waves

Significant wave height may be computed on the basis of the wave height measurement, as a mean value from the 1/3 highest registered wave amplitudes. It can also be estimated based on the formula:

(20) $$H_{1/3est}=4.00\sqrt{D_{\zeta }^{2}},$$

where $D_{\zeta }^{2}$—measured wave height variance. Measured and estimated significant wave heights are compared in Table 4. They are supplemented with their relative estimation error. This error does not exceed 14% and reaches its maximum value for the lowest waves.

The results obtained for the convergence of significant wave height estimations and measurements indicate that it is reasonable to conduct further wave analysis using standard formulas of wave heights, energy and spectra.

Table 1 presents sea state codes (SSCs) and the relevant wave heights for the fully developed sea. These figures were augmented in Table 5 with corresponding lake wave heights and wind force indicators, scaled to the ship dimensions. The lines marked in bold correspond to the values confirmed in the performed experiments, whereas the wave heights in regular font are the direct estimates.

As one can be seen in the last column of Table 5, rescaled lake winds covering the full range of BFT are able to generate waves with heights corresponding to SSC 5–6 only, while in the measurements, maximal wave heights corresponding to SSC of 3 (slight) were recorded (marked in bold in Table 5). Sea states equal to or above SSC 6 require winds of extremely high speeds comparable to hurricanes, so they remain outside the scope of this research.

**Figure 9:**
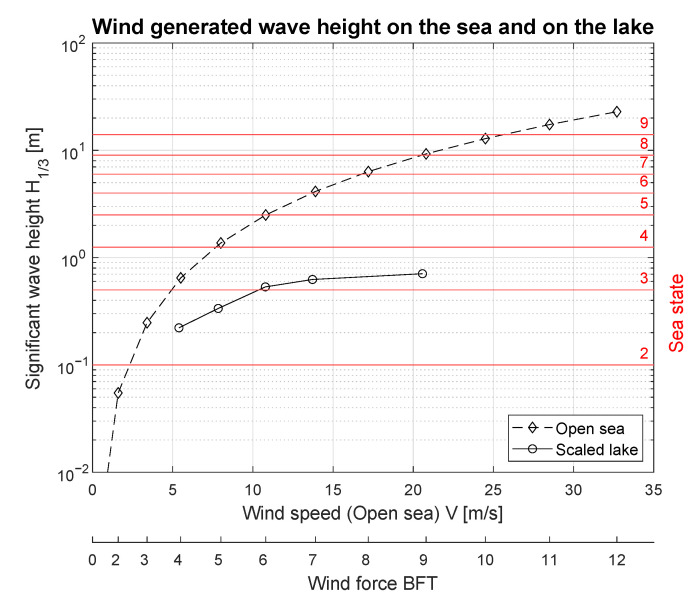
Significant wave height for the fully developed sea and for the lake as a function of the wind force in BFT.

Figure 9 demonstrates this relationship between the wind speed and significant wave height. The ‘open sea’ data refer to significant theoretical wave heights (Table 5) generated by the wind, and ’scaled lake’ data illustrate real measurements presented numerically in Table 4. Sea states, according to the SSC, are indicated by the horizontal red lines. Silm Lake wind speed and wave significant height measurements were scaled by the respective $\sqrt{su}=\sqrt{24}$ and su=24 factors to obtain the wind–wave characteristics in a ship scale comparable to open sea quantities. Lake wave height measurements were retrieved for the ship scale model for sea states ranging from SSC 1 to 4. Due to the size of the lake area and thus inability to register fully developed waves, the wind–wave curve is more flat than corresponding open sea one. In the lake conditions, a rescaled wind speed of 20 m/s corresponds to the SSC 4 sea state, whereas in the open sea environment the same wind force raises SSC 7 waves.

Having estimated the significant wave heights, the wave statistics, including deviations, amplitudes and their distributions, were collected. They are presented and described in Section 4.3.

### 4.3. Measurements and Wave Statistics

**Figure 10:**
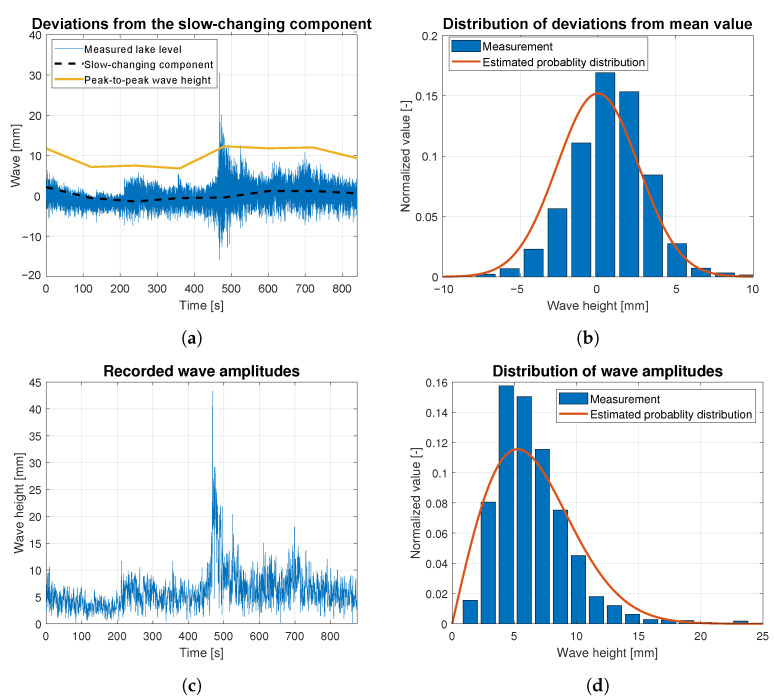
Statistical analysis of the wave height corresponding to 4 BFT wind force in ship scale: (**a**) deviations from the slow-changing component; (**b**) probability distribution of wave height deviations from mean value; (**c**) recorded wave amplitudes; (**d**) probability distribution of wave height amplitudes.

**Figure 11:**
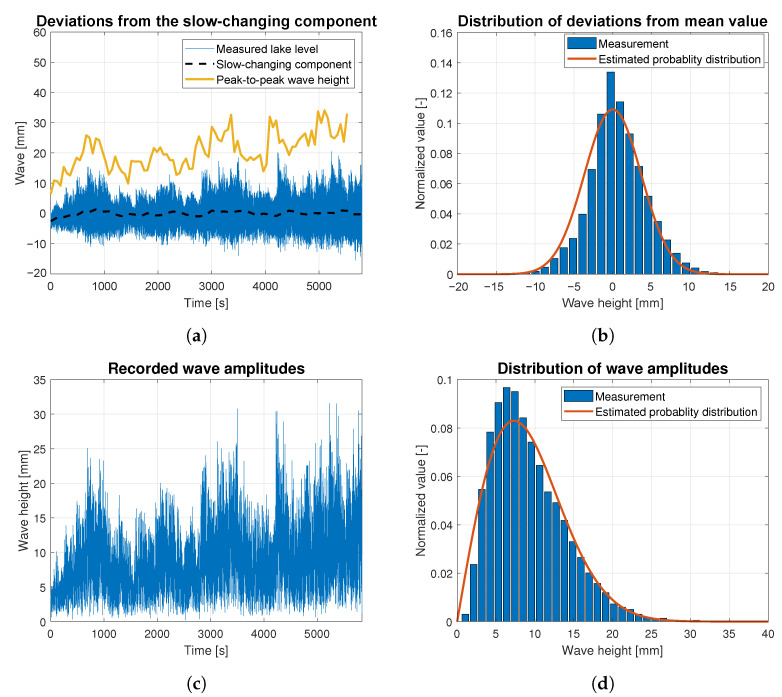
Statistical analysis of the wave height corresponding to 5 BFT wind force in ship scale: (**a**) deviations from the slow-changing component; (**b**) probability distribution of wave height deviations from mean value; (**c**) recorded wave amplitudes; (**d**) probability distribution of wave height amplitudes.

**Figure 12:**
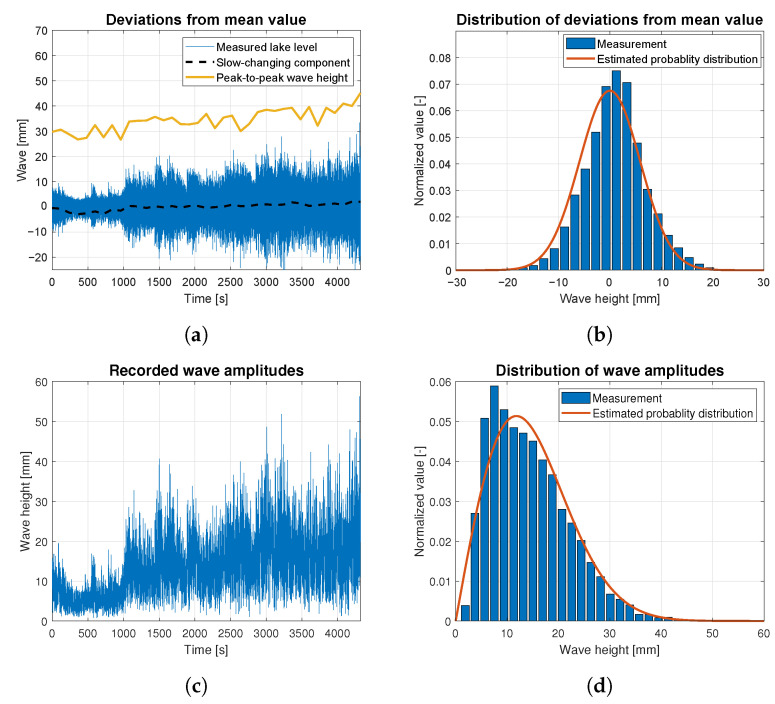
Statistical analysis of the wave height corresponding to 6 BFT wind force in ship scale: (**a**) deviations from the slow-changing component; (**b**) probability distribution of wave height deviations from mean value; (**c**) recorded wave amplitudes; (**d**) probability distribution of wave height amplitudes.

**Figure 13:**
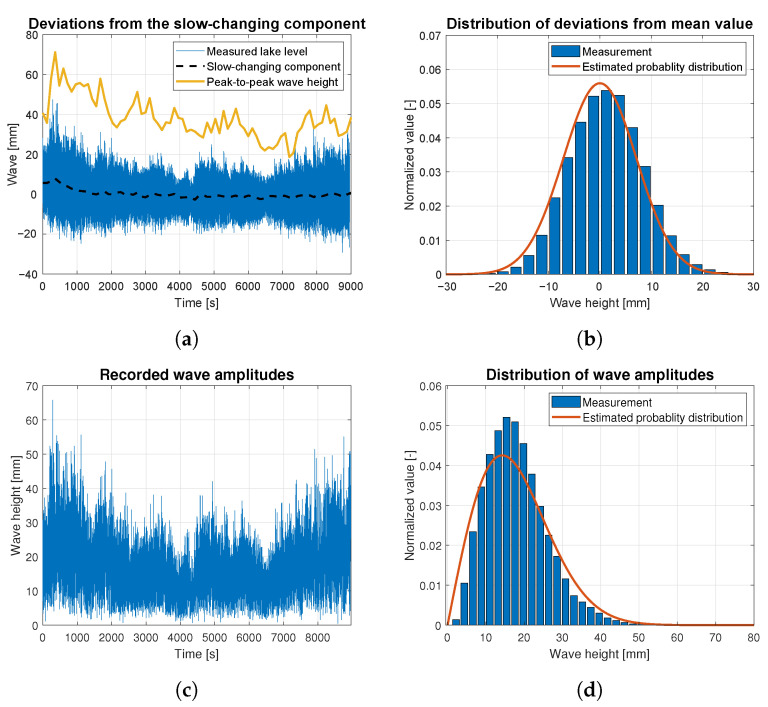
Statistical analysis of the wave height corresponding to 7 BFT wind force in ship scale: (**a**) deviations from the slow-changing component; (**b**) probability distribution of wave height deviations from mean value; (**c**) recorded wave amplitudes; (**d**) probability distribution of wave height amplitudes.

**Figure 14:**
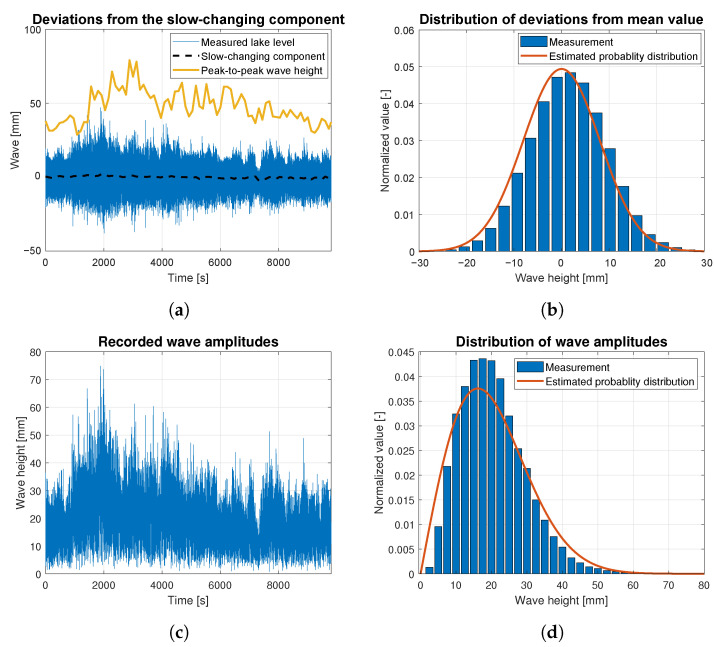
Statistical analysis of the wave height corresponding to 9 BFT wind force in ship scale: (**a**) deviations from the slow-changing component; (**b**) probability distribution of wave height deviations from mean value; (**c**) recorded wave amplitudes; (**d**) probability distribution of wave height amplitudes.

Wave data statistics were determined based on the raw data measurements for all data batches related to wind forces ranging from 4 to 9 BFT. They are presented as the time-dependent functions of wave height deviations from the slow-changing component in Figure 10a, Figure 11a, Figure 12a, Figure 13a and Figure 14a. The slow-changing component was estimated as an average water level every 120 s. Peak-to-peak wave height in these figures corresponds to averaged wave amplitudes computed every 120 s. In order to extract wave statistics and recorded amplitudes, the slow-changing component was filtered out from the raw data before further analysis. Figure 10c, Figure 11c, Figure 12c, Figure 13c and Figure 14c illustrate the processed raw data for computing wave amplitudes using the wave zero-crossing method. Figure 10b, Figure 11b, Figure 12b, Figure 13b and Figure 14b and Figure 10d, Figure 11d, Figure 12d, Figure 13d and Figure 14d present normalized wave height deviations from mean value and subsequent distributions of amplitudes. They both are compared to estimated probability density functions, which are appropriately described by Gaussian and Rayleigh distribution functions [30]. These probability distribution functions are indicated by the red lines and compared with normalized histograms representing raw data statistical attributes.

Figure 10 illustrates the statistical analysis of waves generated by the wind of 4 BFT force in ship scale. The black dashed line in Figure 10a shows that very slow lake level changes generated by the wind are observed. These fluctuations are similar in shape to a sine wave with an amplitude of approx. 4 mm. Between 450 and 500 s, an increase in wave height is observed in Figure 10a,c. This manifests as additional height of almost 0.16 units for the bar in the histogram plot, presented in Figure 10d, which should not appear when comparing to the Rayleigh distribution function. In Figure 10b, the highest bar of the histogram is shifted to the higher amplitude waves, to the right of relative zero value. The histogram shape is similar to that expected for Gaussian distribution. This shift is related to the increase of water level in the lake at the measurement site, due to long-lasting wind speed increase, which can be observed in Figure 10a.

Figure 11 presents the statistical analysis of waves generated by the wind of 5 BFT force in ship scale. The slow-changing wave component is indicated by the dashed black line in Figure 11a. This slow-changing component is sinusoidal with an amplitude of 3 mm. Peak-to-peak mean wave height (indicated by yellow solid line) is increasing, due to long-lasting wind of a 5 BFT force. See Figure 5a. Both histograms presented in Figure 11b,d show convergence with appropriate probability distributions marked by red lines.

Figure 12 presents statistics of the waves generated by the wind of 6 BFT force in ship scale. During this measurement waves slow-changing component was increasing monotonically and sinusoidal change similar to these presented in Figure 10c and Figure 11c, was not observed. Data drawn in Figure 12 were registered during experiment lasting more than 4000 s, in which wind force and direction were stable. Due to water level increase in the measurement area, in the graph of probability distribution of the deviations from mean value (Figure 12b), a shift in the histogram toward higher wave amplitudes was observed. This is a result of the nonlinear mean water level increase. Figure 12c presents the wave development process over time induced also by the significant average wind speed increase depicted in Figure 6a. The Silm Lake waves have developed from the level of 10 mm to the highest amplitudes reaching 60 mm for about 80 min.

Figure 13 illustrates statistical analysis of the waves height induced by the wind of 7 BFT force in ship scale. The Slow-changing component presented in Figure 13a is monotonically decreasing from 7 mm in 500 s of the experiment to values oscillating around 0 mm after 5000 s of the trial. Recorded oscillations are of sinusoidal shape, similar to the results presented in Figure 10a and Figure 11a. Their amplitude reaches to 3 mm. Frequency of the wave height deviations from mean value (Figure 13b) is Gaussian distributed and fits into estimated probability density curve, whereas the distribution of wave amplitudes has a shape similar to the Rayleigh distribution, except the amplitudes of the highest bars of the histogram, which exceed the estimated probability distribution by 10% points. This means that up to 10% more waves with amplitudes exceeding the theoretically expected values by 10 to 25 mm were observed. Similar phenomena were noticed in the case of lower waves generated by the winds of 4–5 BFT in the ship scale. In these cases, however, only 8% and 2% more waves had higher amplitudes than expected.

Figure 14 displays the statistical evaluation of the wave heights generated by the wind of 9 BFT force in ship scale. The low-frequency component in the lake level measurements was very weak (Figure 13a). This signal oscillates only about 1–2 mm from zero level. During measurement lasting about 2.5 h, an average amplitude change from 20 to 40 mm was observed in the first phase of the experiment, while after 50 min, the wave amplitude has stabilized (Figure 14c). The probability distribution of the deviations from the slow-changing wave component value, presented in Figure 14b, has a Gaussian character and fits the estimated probability density distribution. However, the density distribution of wave amplitudes is characterized by higher values than expected. This disproportion is, however, relatively small and only 1% more waves have amplitudes higher by about 15 to 25 mm than expected.

The results of the measured data processing summarized in this subsection show that wave height deviations from mean value and wave amplitude distributions are analogous to the probability distributions, described in the reference literature. Wave height correlation with the wind force is also observed. Thus, the wave amplitude grows together with the wind speed increase.

According to the results presented in Section 4.3 of wave height distributions and statistics, the ‘lake’ wave is similar in nature to the ‘sea’ wave. Therefore a ‘sea’ wave spectra modeling method was adopted for the ‘lake’ case. The results of this research are presented in Section 4.4.

### 4.4. Measured Wave Spectra Modeling

According to the results presented in Section 4.1 and Section 4.3, phenomena on the Silm Lake represent undeveloped wind-induced waves. Therefore, taking into account the literature review, an attempt was made to match the empirically developed wave spectra with the standard spectra.

**Figure 15:**
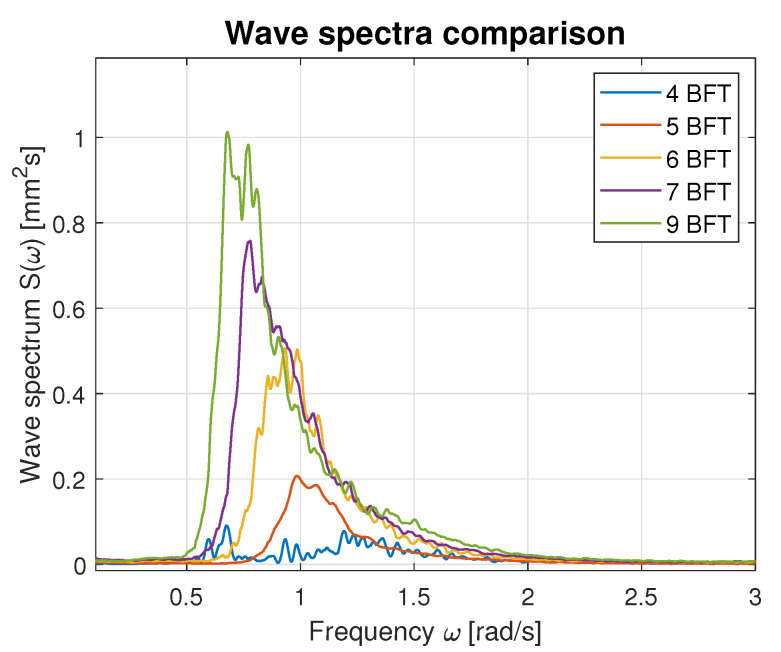
Empirical wave spectra.

Figure 15 presents elaborated wave spectra for all data chunks in the one graph. A shift of the spectrum maxima toward lower frequencies can be observed as the wind speed increases. Furthermore, increase of the wave spectrum values together with the wind speed increase is observed. Qualitatively, this wave empirical spectra chart is comparable to the graph of the ITTC spectrum [23]. Based on the observed similarities, an attempt was made to rescale the ITTC spectrum to match the measured spectra.

Due to the way in which measurement data was analyzed in Section 4.3, the natural wave parameters that are described are significant wave height and the period. Thus, it was decided to conduct a comparison with the ITTC spectrum parameterized by mean significant wave height and period given by Equations (12) and (13). Finally, it was established that parameters *A* and *B* are to be scaled by 0.0226 and 0.1526, respectively.

(21) $$A=3.91{\bar{H}}_{1/3}^{2}/{\bar{T}}_{z}^{4}$$

(22) $$B=105.44{\bar{T}}_{z}^{4}$$

**Figure 16:**
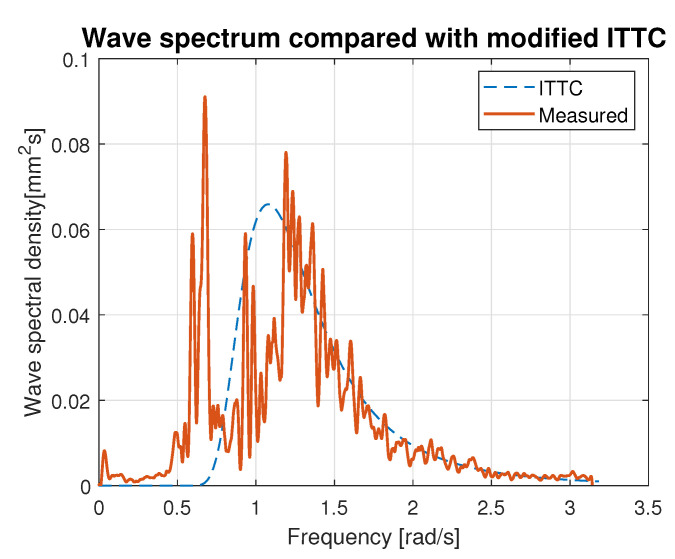
Comparison of the empirical wave spectrum evaluated for 4 BFT wind force in ship scale and the modified ITTC model.

**Figure 17:**
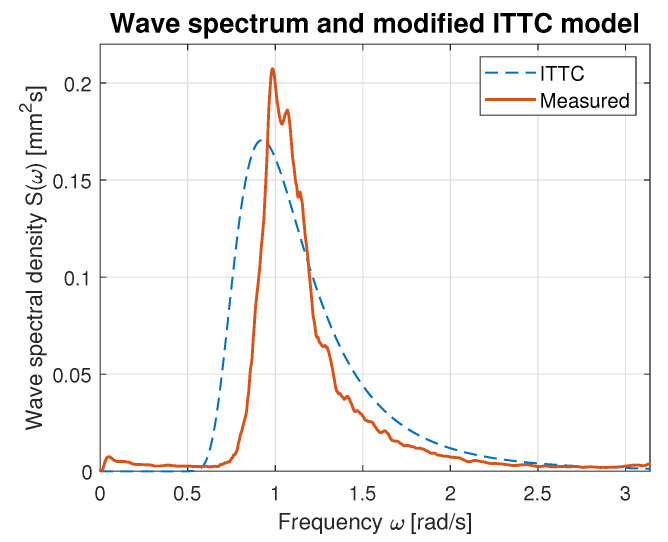
Comparison of the empirical wave spectrum evaluated for 5 BFT wind force in ship scale and the modified ITTC model.

**Figure 18:**
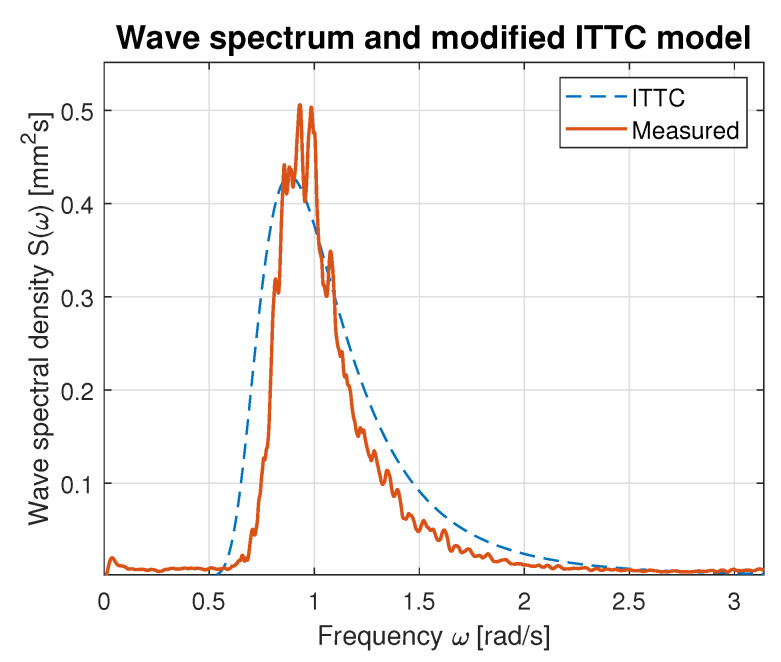
Comparison of the empirical wave spectrum evaluated for 6 BFT wind force in ship scale and the modified ITTC model.

**Figure 19:**
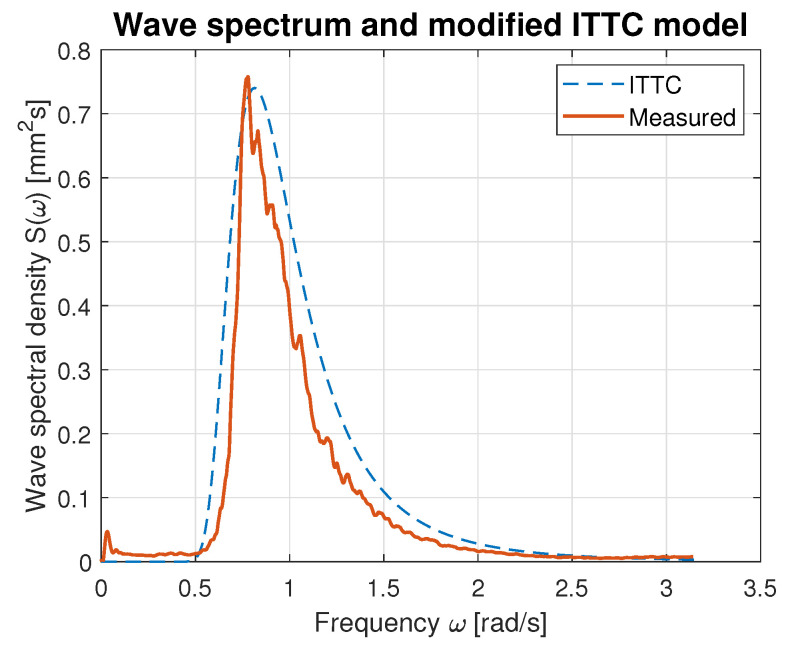
Comparison of the empirical wave spectrum evaluated for 7 BFT wind force in ship scale and the modified ITTC model.

**Figure 20:**
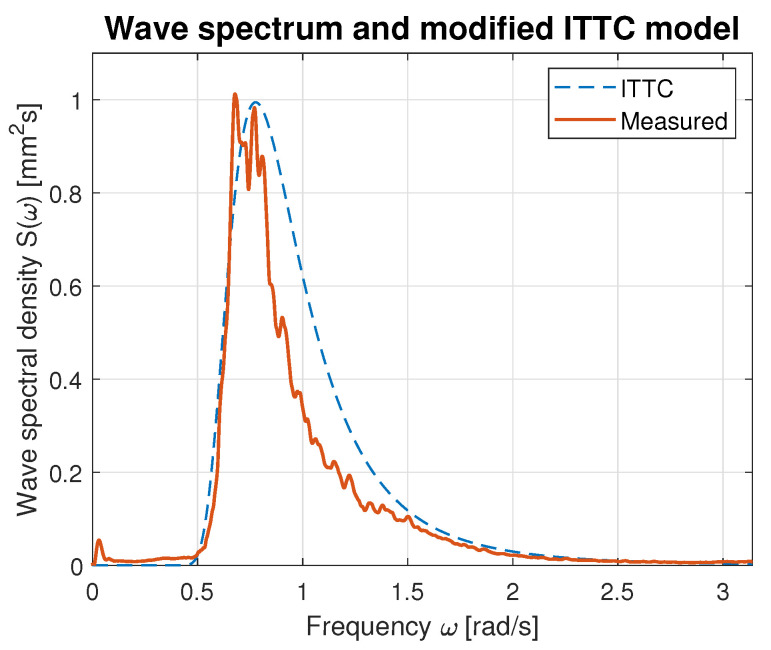
Comparison of the empirical wave spectrum evaluated for 9 BFT wind force in ship scale and the modified ITTC model.

Hence, the values of parameters *A* and *B* calculated using Equations (21) and (22) were used for modified ITTC spectrum computation and plotting. Figure 16, Figure 17, Figure 18, Figure 19 and Figure 20 illustrate the qualitative wave spectrum fit to the modified ITTC spectrum.

In Figure 16, the spectrum for the lowest measured waves is presented. This function is tightly frayed and has two peaks located at 0.5 and 1.2 rad/s. In other cases, shown in Figure 17, Figure 18, Figure 19 and Figure 20 obviously better matching is observed, and the characteristics are smoother.

To estimate quantitative similarity between measured and modified ITTC spectrum their root mean square error (*RMSE*) was calculated according to the formula:

(23) $$RMSE=\sqrt{\sum_{k=1}^{n}\frac{\left[\hat{S}\left(f_{k}\right)-S\left(f_{k}\right)\right]}{n}},$$

where $\hat{S}\left(f_{k}\right)$—observed wave spectrum; $S(f_{k})$—estimated wave spectrum.

Table 6 summarizes measured spectrum fit to the modified ITTC spectrum. The *RMSE*s are presented for all analyzed wind forces in the ship scale. Moreover, comparison of the peak frequencies of both measured and estimated spectra are presented together with their modal values. Quantitative analysis shows an increase in the *RMSE* with the rise of wind force and wave height increase. However, it does not exceed 10% of the spectrum modal value. Differences in the estimated and measured peak frequencies do not exceed 0.1 rad.

## 5. Discussion

The results of the experiments reveal that there is a possibility to rescale one of the standard, known, and widely described in the literature wave spectrum to model the irregular wind-induced waves on the small inland water area such as Silm Lake. Indeed, wind wave signal generation scheme on the lake is similar to the one used to characterize sea waves. It may be described by the aggregation of two components, namely slow-changing and time-varying (WF—wave frequency signal). The slow-changing component is related to the water mass movement within the lake area. The manifestation of the fast-changing component is related to the snatching of water surface molecules by the wind. This is the mechanism of wind wave formation. In this process, there is high correlation between wind speed and wave height. The results shown in Section 4.1 demonstrate that waves generated on the Silm Lake have high correlation factor with the wind speed. The nature of the slow-varying component, however, is related to the direction of waves propagation. This mechanism is known from theory and has not yet been studied on the surveyed lake. This may be a future direction of research.

SSC and their corresponding sea wave heights were compared with their counterparts scaled to the dimensions of the model ship. This comparative analysis showed that the influence of the wave action on the model ship hull going on the lake is significantly smaller than of the waves impacting on a merchant ship operating at sea. This phenomena is related to the ratio of the wind wave height to the ship’s freeboard. In sea conditions, this factor is much larger. Furthermore, in the regular conditions, corresponding to scaled wind in BFT ranging from 0 to 12, there is a possibility to register scaled waves corresponding to the scaled SSC ranging only from 0 (calm) to 6 (very rough). Naturally, there exists a possibility to register higher waves on the Silm Lake, especially during autumn and winter seasons, but they correspond to the scaled hurricane winds in which sailing and especially tests of automatic control of ship motions makes a little sense. Therefore, these conditions and potentially generated environmental disturbances remained beyond the focus of this research.

The height of wind waves occurring on the lake are low, even when they are measured at the ship scale. Therefore, their impact on the ship moving half- or full-ahead is slight. The goal of this research was to estimate the wave spectrum, which may then be used during control system design verification and in the simulations, where the effect of external disturbances have to be taken into account. This model will be useful particularly in the development of ship dynamic positioning (DP) systems, control of precise, slow motion, or ship-to-ship replenishment operations. In these cases, even small external forces should be included in the models because their influence on the ship cannot be neglected. Similarly, these small amplitude waves have significant impact on the small crafts or unmanned surface vehicles (USV) moving over the lake. These types of vessels and their control systems are the subject of a growing number of ongoing studies on autonomous systems. To date, the authors have not found research results of modeling wave-induced disturbances on the open to the natural wind action, confined waters, such as lakes. Presented research results have not in-depth character and may be used as a reference for more accurate lake wave disturbances modeling.

On the other hand, during the wind-induced wave analysis, it turned out that there are great similarities of the examined phenomenon nature to sea waves. Distribution of the wave height deviation from the mean value is Gaussian and wave amplitudes exhibit Rayleigh distribution. The same probability density distributions are obtained for sea waves, as described in the literature [30]. Moreover, measured significant wave heights and those corresponding to them, but estimated using wave height variance differ by no more than 14% of the measured wave height. This conformity between measurements and calculations shows that the nature of lake and sea waves from the perspective of the ship load modeling is very similar.

## 6. Conclusions

The outcome of the research presented in this paper has shown that wind-generated irregular waves on a small lake can be modeled using the slightly modified standard ITTC type spectral density function. However, due to very limited fetch, the amplitudes of the waves are clearly lower than for open sea waves. The purpose of this search for lake surge representation was a need for environmental load modeling for the control systems of a scale model ships tested on this lake. Even after correction of the measurements according to scale factor of the ship models, the waves are lower than corresponding ones at the open sea. Winds up to the force of 9 BFT in model scale can induce the waves in the model scale up to only SSC 4.

Because the wave model obtained in the research fits the standard spectral representation, it is easy to apply it in the simulation and verification tools for ship motion control systems. However, the limited range of reproducible wave heights limits its use to disturbance-sensitive applications.

The research is limited in scope. The measurements of the waves were conducted in one location. The direction of the waves propagation was not evaluated. The test areas are only accessible in summer, and measurements of waves for strong storm winds were therefore not available in the datasets. Supplementing the data to overcome these limitations seems to be a natural direction for further development of the research. The verification of findings of this project based on the data collected on different water area of similar character may be another interesting area to explore.

## Figures and Tables

**Table 1:** Sea state codes [20].

Code	Description	$H_{1/3}$ [m]	Percentage of Occurrence		
			World Wide	North Atlantic	Northern North Atlantic
0	Calm (glassy)	0.00			
1	Calm (rippled)	0.00–0.10	11.2486 ^1^	8.3103	6.0616
2	Smooth (wavelets)	0.00–0.50			
3	Slight	0.50–1.25	31.6851	28.1996	21.5683
4	Moderate	1.25–2.50	40.1944	42.0273	40.9915
5	Rough	2.50–4.00	12.8005	15.4435	21.2383
6	Very rough	4.00–6.00	3.0253	4.2938	7.0101
7	High	6.00–9.00	0.9263	1.4968	2.6931
8	Very high	9.00–14.00	0.1190	0.2263	0.4346
9	Phenomenal	over 14.00	0.0009	0.0016	0.0035

^1^ Percentage for codes 0, 1 and 2 is summarized.

**Table 2:** Data of sensor in static characteristic linearity verification.

Immersion Depth Δh [mm]	Digital Value DV	Fit	Fit Error	
			Absolute [–]	Relative [%]
0.0	43,790	43,941	−150.78	−0.34
−16.5	42,380	42,357	22.67	0.05
−38.5	40,200	40,246	−46.05	−0.11
−60.5	38,130	38,135	−4.78	−0.01
−95.0	34,760	34,824	−43.92	−0.13
−115.0	33,330	32,857	473.40	1.42
−133.5	31,230	31,129	100.81	0.32
−155.5	29,190	29,018	172.08	0.59
−183.0	26,100	26,379	−278.82	−1.07
−199.5	24,590	24,795	−205.37	−0.84
−221.5	22,520	22,684	−164.10	−0.73
−243.5	20,470	20,573	−102.82	−0.50
−273.0	17,870	17,742	128.20	0.72
−295.0	15,730	15,631	99.48	0.63

**Table 3:** Statistical analysis of wave measurement disturbances.

Mean Digital Value	Min. Digital Value	Max. Digital Value	Standard Deviation σ	Max Discrepancy of Water Level Measurement Δh [mm]
43,790	43,710	43,880	26.32	1.77
42,380	42,300	42,460	28.52	1.67
40,200	40,100	40,280	28.73	1.88
38,130	38,040	38,260	29.5	2.29
34,780	34,690	34,880	26.92	1.98
33,330	33,260	33,420	29.22	1.67
31,230	31,160	31,300	27.82	1.46
29,190	29,100	29,270	29.05	1.77
26,100	26,040	26,150	19.02	1.15
24,590	24,520	24,660	24.39	1.46
22,520	22,470	22,560	17.65	0.94
20,470	20,410	20,550	20.42	1.46
17,870	17,810	17,910	14.57	1.04
15,730	15,670	15,780	13.93	1.15

**Table 4:** Measured and estimated significant wave height comparison.

BFT in Scale Ship	Estimated Significant Wave Height $H_{1/3est}$ [mm]	Measured Significant Wave Height $H_{1/3}$ [mm]	Significant Wave Height Error [%]	Measured Wave Height Variance $\left(D_{\zeta }^{2}\right)$
4	10.50	9.24	13.65	6.89
5	14.62	14.00	4.42	13.37
6	23.62	22.23	6.26	34.88
7	29.05	26.07	11.42	52.75
9	32.30	29.50	9.48	65.20

**Table 5:** Sea state codes and corresponding scaled lake wave heights and wind forces.

Sea State Code	Description	Open Sea Wave Height $H_{1/3}$ [m]	Scaled Lake Wave Height $H_{1/3}$ [mm]	Corresponding Scaled Lake Wind Force [BFT]
0	Calm (glassy)	0.00	0.00	0
**1**	**Calm (rippled)**	0.00–0.10	**0.00–4.2**	**0–3**
**2**	**Smooth (wavelets)**	0.00–0.50	**0.00–20.8**	**3–6**
**3**	**Slight**	0.50–1.25	**20.8–52.0**	**6–9**
4	Moderate	1.25–2.50	52.0–104.2	9–10
5	Rough	2.50–4.00	104.2–166.7	10–11
6	Very rough	4.00–6.00	166.7–250.0	12
7	High	6.00–9.00	250.0–375.0	–
8	Very high	9.00–14.00	375.0–583.3	–
9	Phenomenal	over 14.00	over 583	–

**Table 6:** Evaluation of the empirical spectra fit to the modified ITTC standard model.

BFT in Ship Scale	*RMSE*	Estimated Peak Frequency $f_{\mathrm{pest}}$ [rad]	Observed Peak Frequency $f_{p}$ [rad]	Max. Estimated Spectrum Value $\left[{\mathrm{mm}}^{2}s\right]$	Max. Observed Spectrum Value $\left[{\mathrm{mm}}^{2}s\right]$
4	0.02	1.07	1.19	0.07	0.08
5	0.03	0.90	1.00	0.17	0.20
6	0.05	0.89	0.93	0.42	0.50
7	0.07	0.83	0.78	0.74	0.76
9	0.10	0.78	0.69	0.99	0.99

## Data Availability

Not applicable.

## Abbreviations

The following abbreviations are used in this manuscript:

BFT	Beaufort wind scale
DP	dynamic ship positioning
ITTC	International Towing Tank Conference
JONSWAP	Joint North Sea Wave Project
PEF	peak enhancement factor
PM	Pierson–Moskowitz spectrum
PSD	power spectral density
RAO	response amplitude operator
RMSE	root mean square error
SSC	sea state code
SWAN	Simulation of WAves in Near shore areas
USV	unmanned surface vehicle
WF	wave frequency
WW3	WaveWatch III
